# Antipyrin a Hemostatic

**Published:** 1892-08

**Authors:** W. M. Powell

**Affiliations:** Albany, Texas


### ANTIPYRIN A HEMOSTATIC.

ALBANY, TEXAS, August 3, 1892.

*Editor Daniel's Texas Medical Journal:*

I see in the July number of your valuable journal that Dr. F. C. Ford, of Nacogdoches, Texas, is given credit for priority in the discovery of antipyrin as a styptic. If you will look up a short article of mine in the March number of your journal of 1888, page 366, you will see that I am the first one in this country to use it, and that it was not original with me. I have since publishing the paper referred to, employed the same agent in minor operations, with like good results.

Truly yours,

W. M. POWELL, M. D.

